# Canadian supportive care recommendations for the management of anemia in patients with cancer

**DOI:** 10.3747/co.2007.149

**Published:** 2007-10

**Authors:** J. Mikhael, B. Melosky, C. Cripps, D. Rayson, C.T. Kouroukis

**Affiliations:** * Princess Margaret Hospital, Toronto, Ontario; † BC Cancer Agency, Vancouver, British Columbia.; ‡ Ottawa Hospital Regional Cancer Centre, Ottawa, Ontario.; § QEII Health Sciences Centre, Halifax, Nova Scotia.; || The Juravinski Cancer Centre, Hamilton, Ontario

**Keywords:** Canadian recommendations, anemia, cancer, supportive care, erythropoiesis-stimulating agent, esa, chemotherapy-induced anemia, cia, iron, safety

## Abstract

Anemia is a common finding in cancer patients, most often as a result of chemotherapy. Management of anemia requires a comprehensive approach of appropriate diagnosis, exclusion of reversible causes, use of erythropoiesis-stimulating agents (esas), and iron supplementation. Recently, consensus guidelines on the management of chemotherapy-induced anemia were published in Europe and the United States. The present review is intended to be a practical guide for Canadian physicians, based on published guidelines, but specifically tailored to the Canadian environment.

Recommendations for the use of esas are presented, including initiation, target hemoglobin, dosing and adjustments, monitoring, and re-initiation. Issues of safety are also addressed, including thromboembolic risk, impact on survival, and tumour progression. The importance of iron metabolism and the use of iron supplementation (both oral and parenteral) is discussed.

## 1. INTRODUCTION

Anemia is common in cancer 1 and may be the result of many factors, including chemotherapy, marrow infiltration, radiation, nutritional deficiencies, and blood loss. Anemia in cancer adversely affects the patient by producing fatigue, cognitive impairment, a need for blood transfusions, and possible disruption of therapy. It is therefore incumbent on the treating physician to address anemia in cancer.

Easily reversible causes such as nutritional deficiencies, hypothyroidism, and iron deficiency should be corrected immediately; however, these causes account for only a small proportion of anemias in cancer patients.

One method of treating anemia (in addition to transfusion) is the use of an erythropoiesis-stimulating agent (esa). The rationale for the use of esas in chemotherapy-induced anemia (cia) has been twofold: to improve the patient’s hemoglobin level and to reduce the need for transfusions. A multitude of clinical trials and meta-analyses have validated the use of esas for these two purposes. In addition, the data have also demonstrated improvements in quality of life contributing to the indication for the use of esas in Canada 2–6.

The purpose of the present article is to use published data and guidelines and consensus opinion from Canadian physicians to provide recommendations for the treatment of cia in Canada. The recommendations provided here focus specifically on the use of esas for cia.

A medical guideline aims to guide decisions and criteria in specific areas of health care by examining and synthesizing the best current evidence. Guidelines can have a significant economic impact in health care and can also provide appropriate access to new medications. Patients benefit from guidelines by receiving timely treatment based on supporting evidence and also better information that facilitates treatment choice. Health care professionals receive new recommendations based on current clinical evidence, which also supports quality improvement activities. As a result of guidelines, health care systems may improve their efficiency and optimize value for money.

## 2. DISCUSSION

### 2.1 Existing Guidelines

The American Society of Clinical Oncology / American Society of Hematology (asco/ash), the European Organization for Research and Treatment of Cancer (eortc), and the National Comprehensive Cancer Network (nccn) have all published guidelines for the use of esas in patients with cancer 7–9. Variations between the guidelines can be attributed primarily to the scientific literature available at the time of guideline development 10:

- The 2002 asco/ash guidelines 7 were based on a U.S. Agency for Healthcare Research and Quality topic review covering the literature on esas published between January 1985 and October 1999. During that period, epoetin was the only esa available.
- In 2004, eortc published its guidelines for the use of esas in patients with cancer and cia 8. These guidelines were rooted in literature published from 1996 through 2003 and included a review of epoetin alfa, epoetin beta, and darbepoetin alfa. In 2006, the guidelines were updated using literature published through November 2005 11.
- The nccn guidelines for the management of cia^9^ are the most recent, having been published in 2007. The guidelines are based on a panel review of an updated meta-analysis in the Cochrane Database of Systemic Reviews on the use of esas in patients with cancer 6.

### 2.2 Canadian Recommendations

#### 2.2.1 ESA Treatment Goals and Targets

The goals of esa management of symptomatic anemic patients with malignancy undergoing systemic chemotherapy are to maximize quality of life and minimize transfusion requirements while using the lowest dose of esa necessary to achieve a target hemoglobin level.

International guidelines vary slightly in their recommendations for target hemoglobin level. The asco/ ash and nccn hemoglobin target is 120 g/L; eortc recommends a target range of 120–130 g/L. Product monographs for epoetin alfa and darbepoetin alfa both indicate that target hemoglobin should not exceed 120 g/L, with a rate of rise not greater than 10 g/L over a 2-week period 12, 13.

Notably, a hemoglobin target is a designated value *not to be exceeded,* rather than a target to be aimed for. Hemoglobin levels higher than 130 g/L are not recommended, because randomized clinical trials have observed an adverse impact on overall survival at such levels 14.

#### 2.2.2 ESA Dosing and Adjustments

Optimal esa dosing and titration for both epoetin alfa and darbepoetin alfa for patients with cancer receiving systemic chemotherapy have been well established through randomized trials.

##### Initiating ESA Dose

**FIGURE 1:**
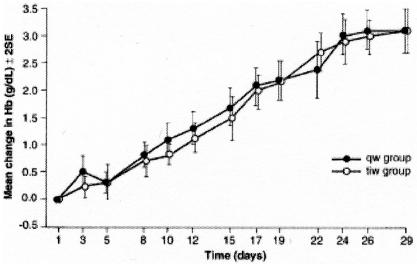
Profile of mean change in hemoglobin (g/dL) from baseline after initiation of epoetin alfa, for three times weekly and weekly dosing regimens 15. Used with permission of Springer Berlin.

**FIGURE 2:**
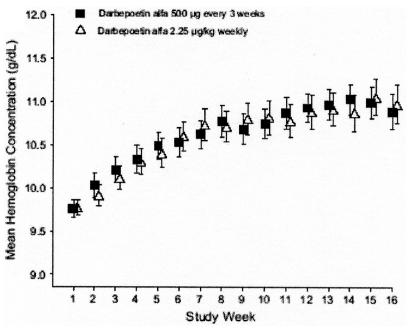
Mean hemoglobin concentration (g/dL) after initiation of darbepoetin alfa, for weekly and every-3-week dosing regimens 16. Used with permission of Oxford University Press.

In adults, epoetin alfa and darbepoetin alfa each have two recommended initiation dosing schedules (Table I). Titration graphs illustrate the mean change in hemoglobin concentration from initiation of epoetin alfa (Figure 1) and mean hemoglobin concentration levels for darbepoetin alfa (Figure 2) using various dosing regimens 15, 16.

##### Initiating Hemoglobin Level

The Canadian standard has been to initiate esa therapy at a hemoglobin level below 110 g/L in symptomatic patients. As with recommendations on hemoglobin targets, recommendations on esa initiation vary slightly between published guidelines (Table II) The guideline from nccn 9 recommends consideration of an esa when the patient’s hemoglobin level is below 110 g/L. The guideline from eortc 8 initially recommended esas for either asymptomatic or symptomatic cancer patients with hemoglobin levels of 90–110 g/L who are receiving chemotherapy. In a 2006 update, eortc refined the guideline by stating that “esas may be considered in asymptomatic patients with hemoglobin levels below 119 g/L to prevent a further decline according to individual characteristics” 11 The guideline from asco/ ash 7 recommends esas for patients with hemoglobin levels below 100 g/L who are receiving chemotherapy and, if symptomatic, for patients with hemoglobin levels below 120 g/L.

Some common principles emerge from the various guidelines—for example, a consideration of patient characteristics such as baseline hemoglobin level, rate of decline of hemoglobin, number of chemotherapy cycles remaining, and clinical symptomatology when initiating esa therapy.

It is important to note that initiation of esas should not be delayed. A meta-analysis by Couture *et al.*^17^ showed that early treatment with epoetin alfa could reduce the number of red blood cell transfusions in patients receiving chemotherapy. Quirt *et al.* 18 further demonstrated that the initiation of esas at a hemoglobin level below 100 g/L resulted in a higher risk of blood transfusions. Finally, a systematic review by Lyman and Glaspy 19 found a reduction in the relative risk of transfusion and hemoglobin below 100 g/L after early intervention (*p* < 0.0001). Collectively, these findings suggest that optimal benefit from esa treatment of cia may be achieved through early intervention.

##### Monitoring

Maintenance therapy requires regular monitoring of hemoglobin levels. For patients who are responding, treatment can continue as long as that response is maintained, tolerance is ongoing, and systemic chemotherapy is continuing (Table III).

##### Dose De-escalation

If the rate of rise or the absolute level of hemoglobin achieved exceeds given values on esa therapy, dose de-escalation should be initiated (Table IV).

##### Non-response and Dose Escalation

Non-response can be defined as the failure to achieve a hemoglobin increase of ≥10 g/L in 4 weeks with weekly epoetin alfa, or in 6 weeks with once-weekly or every-3-weeks darbepoetin alfa. Dose escalation of the esa should be predicated on evidence of non-response and ongoing tolerance to treatment (Table V).

##### Discontinuation

Treatment of anemia with an esa should be discontinued if response is lost or if acute drug toxicities arise. Discontinuation of the esa may be considered in patients who experience a thrombotic event. Considerable controversy remains regarding the optimal continuation time for an esa following completion of chemotherapy. It is reasonable to continue esa therapy for a duration beyond chemotherapy completion equivalent to 1–2 further cycles of therapy—for example, a further 6 weeks for an every-3-weeks regimen).

If, in the epoetin alfa 3-times-weekly dosing schedule, the hemoglobin increase is less than 10 g/L and the reticulocyte count increase is less than 40,000 cells/μL above baseline after a further 4 weeks of therapy at 300 IU/kg, response is unlikely, and treatment should be discontinued. Similarly, for once-weekly dosing, if a satisfactory response to the increased weekly dose of 60,000 IU has not been obtained after a further 4 weeks, then the patient is unlikely to respond, and treatment should be discontinued 12.

##### Re-initiation

Re-initiation of anemia treatment with an esa can be recommended if systemic chemotherapy for malignant disease is again delivered and is accompanied by an observed anemia meeting the same criteria for initiation of esa as during previous chemotherapy cycles. The patient should have no prior history of adverse events associated with esa treatment. It is suggested that the regular criteria for esa initiation, dose adjustment, and discontinuation be applied to all subsequent decisions for esa re-initiation.

#### 2.2.3 Safety of ESAs

Recent data have signalled safety issues involving the use of esas. These concerns—including thromboembolic risk, survival, and tumour proliferation—are reviewed in this subsection.

##### Thromboembolic Risk

An increased risk of thromboembolic events is now felt to be a clearly significant side effect of esas. The recently published Cochrane meta-analysis 6 included 9353 cancer patients enrolled in 57 randomized, placebo-controlled trials conducted from 1985 to 2005 to evaluate epoetin alfa, epoetin beta, or darbepoetin alfa. Treatment with an esa increased the risk of thromboembolic events [relative risk: 1.67; 95% confidence interval (ci): 1.35 to 2.06], with the risk increasing in proportion with target hemoglobin level (Table VI). The cause of thromboembolic events arising in patients with malignancy receiving an esa is complex, arising from a multitude of risk factors, including chemotherapy treatment, presence of metastatic disease, immobility, and the insertion of central venous access devices.

##### Survival Analysis in Clinical Trials

###### Negative Survival

 Four randomized controlled clinical trials of esa therapy for patients with malignancy have demonstrated a negative impact on overall survival 21–23 [Glaspy J, Smith M, Aapro H, *et al.* Results from a phase iii, randomized, double-blind, placebo-controlled study of darbepoetin alfa (DA) for the treatment of anemia in patients not receiving chemotherapy or radiotherapy. Presented at the aacr Annual Meeting 2007; April 14–18, 2007; Los Angeles, CA, U.S.A.].

The Breast Cancer Erythropoietin Survival Trial (best) randomized non-anemic patients with metastatic breast cancer to epoetin alfa or to placebo with a goal of achieving hemoglobin levels of 120–140 g/L 21. Compared with the group receiving placebo, the group receiving the esa had a reduced overall survival rate at 1 year [hazard ratio (hr): 1.35]. This difference became apparent within the first 4 months of therapy initiation and may have been secondary to a higher incidence of thrombotic cardiovascular events.

The enhance trial randomized patients with head-and-neck cancer to placebo or epoetin beta with a goal of achieving hemoglobin levels of 145–150 g/L 22. Patients were not anemic at randomization, and they received concomitant radiation therapy but not chemotherapy. As in the best study, a significantly decreased survival rate was observed in the esa-treated group (hr: 1.39; 95% ci: 1.07 to 1.74). Patients randomized to placebo survived a median of 928 days as compared with 605 days in the epoetin beta group (*p* = 0.09).

The epo-can 20 trial was a randomized, placebo-controlled trial involving anemic patients with non-small-cell lung cancer who were not on active chemotherapy 23. Because of evolving safety concerns regarding esa use in patients not receiving chemotherapy, an unplanned interim safety analysis was performed after 70 patients had been randomized. Median survival rates were higher in the placebo group (hr: 1.84), resulting in study closure—although conclusions were unclear because of the high early mortality rate and the very small sample size.

Amgen’s 20010103 study was a randomized, placebo-controlled trial of darbepoetin alfa for anemic patients with cancer but not receiving chemotherapy [Glaspy J, Smith M, Aapro H, *et al.* Results from a phase iii, randomized, double-blind, placebo- controlled study of darbepoetin alfa (DA) for the treatment of anemia in patients not receiving chemotherapy or radiotherapy. Presented at the aacr Annual Meeting 2007; April 14–18, 2007; Los Angeles, CA, U.S.A.]. The primary end point of transfusion reduction was not met, and an increase in mortality was seen in the esa arm (hr: 1.29). In this preliminary report, median follow-up was only 4.3 months. As in the epo-can 20 study, the use of esas in patients not on chemotherapy has not been common practice and is now discouraged.

Summarizing these four trials, best and enhance treated patients to higher-than-recommended target hemoglobin levels, and the epo-can 20 and Amgen 20010103 trials used esas in cancer patients not on active chemotherapy treatment. The results of these four trials have contributed to the generally accepted recommendations regarding a more modest hemoglobin target of 120 g/L and a restriction of esa use to cancer patients with anemia who are receiving chemotherapy.

###### No Survival Disadvantage

Despite the contrary evidence, some large randomized controlled trials of esa therapy have failed to demonstrate a negative impact on overall survival. Studies involving patients receiving chemotherapy for non-small-cell lung cancer (early versus late intervention) with epoetin alfa 24 and placebo-controlled trials of darbepoetin 25 did not reveal any survival decrement. Another randomized double-blind placebo-controlled trial of patients with small-cell lung cancer demonstrated similar survival rates in epoetin alfa and placebo arms 26.

Recently, another trial (Amgen study 20010145) designed to explore a potential survival advantage when patients with extensive-stage small-cell lung cancer undergoing platinum-plus-etoposide chemotherapy were given darbepoetin alfa was completed. This randomized phase iii placebo-controlled study showed no survival advantage in the darbepoetin arm, with a median survival in both arms of 40 weeks, and a hazard ratio (darbepoetin/placebo) of 0.93 (95% ci.: 0.78 to 1.11) 27

The first Cochrane meta-analysis on this topic included trials through December 2001 and reported inconclusive evidence on the potential impact of esa therapy on overall survival (hr: 0.81; 95% ci: 0.67 to 0.99) 28. The updated Cochrane meta-analysis, incorporating trials up to 2005 (including the best, enhance, and epo-can 20 studies), again concluded with uncertainty regarding “whether and how epoetin or darbepoetin affects overall survival” (hr: 1.08; 95% ci: 0.99 to 1.18) 6.

##### Tumour Progression

Tumour progression secondary to the activation of erythropoietin receptors has been questioned 29. The best and enhance trials both showed reduced tumour control in the setting of targeting hemoglobin levels higher than current usual practice. Another study, dahanca, was modeled after the enhance study to address some of the latter study’s methodologic issues 30.

The dahanca Study was a prospective trial in patients with squamous cell carcinoma of the head and neck who were undergoing treatment with definitive radiotherapy. Patients were randomized to darbepoetin or placebo with a primary endpoint of locoregional control rate at 5 years. An interim analysis of 484 patients demonstrated a 10% increase in the locoregional failure rate among darbepoetin-treated patients (*p* = 0.01). Overall survival was not significantly different, but appeared to trend towards shorter survival in the esa arm (*p* = 0.08). There were limitations to this study, including a lack of uniform imaging assessment at baseline or at recurrence, and a lack of confirmation of recurrent disease by tissue biopsy. The final study analysis will be reported to the U.S. Food and Drug Administration in late 2008.

The relationship between the presence of erythropoietin receptors and tumour proliferation because of exogenous erythropoietin with the use of esas has not yet been firmly established. Likewise, the function of erythropoietin receptors is not well understood. The *in vitro* studies to date vary in their findings and have been called into question based on assessment methodology 31.

##### Safety Conclusions

Safety issues surrounding esas for the treatment of cancer-related anemia include thromboembolic risk, survival, and tumour proliferation. The risk of thromboembolic events are increased with esas, and careful monitoring of hemoglobin levels in patients is mandatory. In May 2004, the U.S. Food and Drug Administration’s Oncologic Drugs Advisory Committee (odac) revised product labelling to include warnings against maintaining hemoglobin levels above 120 g/L. The negative survival studies are difficult to interpret in the context of current practice because of their higher-than-recommended hemoglobin targets, higher-than-recommended doses used, methodologic limitations in the individual trials, and the enrolment of patients not on active chemotherapy. The trials evaluating disease control rates suffer from similar limitations.

Because of the results observed with respect to overall survival in the Amgen 20010103 Study (discussed earlier), odac met again in May 2007. That meeting resulted in the initiation of black box warnings in both Canada and the United States 32. The panel voted to have Amgen and Johnson & Johnson further strengthen the warning labels of their esas and conduct additional safety studies on these agents. The final odac report is pending.

#### 2.2.4 Iron

Despite the availability and ease of administration of esas to treat cia in cancer patients, only about 60% of patients experience an adequate response to such agents 6. Given that one of the pathologic mechanisms in anemia of cancer is the sequestration of iron out of the available circulating pool, and that ample experience exists in nephrology regarding administration of parenteral iron to improve hemoglobin levels in patients with chronic renal failure who are receiving esas, attempts have been made to improve the response to esas in oncology by supplementing with iron.

A few studies have tested iron supplementation in anemic cancer patients receiving chemotherapy and esas. In a study by Auerbach *et al.* 33, cancer patients on chemotherapy who were anemic and receiving weekly epoetin alfa 40,000 IU were randomized to no iron supplementation, oral iron supplementation, total dose intravenous infusion of iron dextran, or weekly intravenous iron dextran. Patients receiving parenteral iron supplementation experienced a greater improvement in their hemoglobin level, and more patients experienced a hemoglobin response (hemoglobin at least 120 g/L or an increase of at least 20 g/L). Quality-of-life analyses suggested that patients treated with parenteral iron experienced improvements in energy, activity, and overall quality of life.

In another study by Vandebroek *et al.* 34, patients receiving chemotherapy for non-myeloid malignancies and being treated with darbepoetin alfa 500 μg subcutaneously every 3 weeks were randomized to oral iron, no iron, or iron sucrose 200 mg intravenously every 3 weeks. Fewer patients required transfusions when treated with intravenous iron. Hemoglobin levels and clinically meaningful improvements in scores on the Functional Assessment of Cancer Therapy–Fatigue appeared higher, but differences were not statistically significant. Iron supplementation was not associated with any significant toxicities. This study is currently available only in abstract form.

In a recently published study by Henry *et al.* 35, patients treated with epoetin alfa at a weekly dose of 40,000 IU were randomized to weekly intravenous sodium ferric gluconate 125 mg, oral ferrous sulphate 325 mg three times daily, or no iron. Hemoglobin increase was significantly better in the intravenous group as compared with the oral iron and no iron groups (24 g/L vs. 16 g/L vs. 15 g/L respectively; *p* = 0.0092 for intravenous as compared with oral iron). Hemoglobin response was seen in 73% of intravenous iron-treated patients as compared with 45% of patients treated with oral iron and 41% receiving no iron.

In another study by Bellet *et al.* 36, cancer patients receiving chemotherapy and esas were randomized to intravenous iron sucrose or to no iron after 8 weeks of esa treatment. All patients continued with esa therapy for up to 12 weeks. Regardless of the response to the initial 8-week course of esa alone, patients receiving iron sucrose experienced a greater hemoglobin rise than did patients not receiving iron.

It appears from the published literature that supplementation with parenteral iron is associated with benefits in patients treated with esas. Hemoglobin level and hemoglobin response both improve, and the frequency of transfusions declines. Parenteral iron appears to be well tolerated, particularly the iron sucrose and sodium ferric gluconate formulations.

Despite the benefits, outstanding questions remain regarding iron supplementation in patients with cia who are receiving esas. Most of the studies have initiated iron supplementation along with an esa, except for one study that tested iron after 8 weeks of esa therapy. No study has compared upfront iron supplementation with no iron supplementation in only poor esa responders. The studies screened patients and did not enrol patients who were felt to be iron-deficient (defined as a serum ferritin level below 100 ng/L and a transferrin saturation below 15%). Patients who are iron-deficient should initially be treated with iron rather than an esa. Regarding the potential for inducing a state of iron overload, the two fully published studies of iron supplementation accepted patients with ferritin levels of up to 675 pmol/L 33 and 900 ng/mL (approximately 2025 pmol/L) 35.

All of the available studies had rather limited periods of follow-up. Potential long-term toxicities of parenteral iron supplementation in cancer patients on chemotherapy have not been reported. No comparative data have been developed to inform the most effective schedule or iron formulation for use in cia patients on an esa. Information is also lacking regarding the potential value of new oral iron compounds (heme iron polypeptide with folic acid or polysaccharide iron, vitamin B complex, and folic acid) in this group of patients.

**FIGURE 3:**
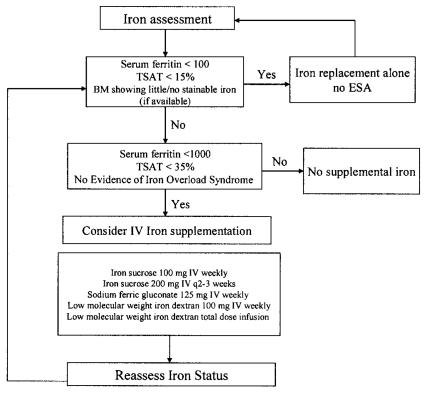
Iron assessment and supplementation in chemotherapy-induced anemia. tsat = transferrin saturation; bm = bone marrow; esa = erythropoietic-stimulating agent; IV = intravenous.

Despite the limited available evidence, the provision of parenteral iron to patients with cia appears to improve response to the esa and represents a reasonable treatment option for physicians to consider for their patients, even for those patients who experience a poor response to an esa. Figure 3 summarizes iron management in patients with cia.

## 3. SUMMARY

Anemia is a common finding in cancer patients, most often as a result of chemotherapy. The management of anemia requires a comprehensive approach of appropriate diagnosis, exclusion of reversible causes, and use of esas and iron supplementation. The ultimate goal of treating cia is to increase hemoglobin levels to reduce the need for transfusions and improve quality of life for the patient. Recommendations for the use of esas include initiation at a hemoglobin level below 110 g/L, a target hemoglobin of 120 g/L, and dose adjustments to stay within appropriate levels. Hemoglobin should be regularly monitored, and rationales for discontinuation of esas or re-initiation should be observed.

Safety issues have recently been raised, namely thromboembolic risk, impact on survival, and tumour progression. Although these mechanisms are not completely understood, strategies to enhance safety include thrombosis prophylaxis when indicated and targeting to appropriate hemoglobin levels. Iron metabolism is critical to anemia, and the use of iron supplementation (especially parenteral iron) may improve response to esas, further reducing the need for transfusions.

## Figures and Tables

**TABLE I:** Initiation of erythropoiesis-stimulating agents (esas)

Epoetin alfa 12 schedules		Darbepoetin alfa^13^ schedules	
Three times weekly	Once weekly	Once weekly	Every 3 weeks
150 IU/kg subcutaneously	40,000 IU subcutaneously	2.25 μg/kg subcutaneously	500 μg subcutaneously

**TABLE II:** A comparison of guidelines for the use of erythropoiesis-stimulating agents (esas) in patients with cancer

	Guidelines		
	asco/ ash^7^	eortc^11^	nccn^9^
esa initiation	Hb≤100 g/L: esa recommended 100 g/L<Hb≤120 g/L: use of esa determined by clinical circumstances	Symptomatic patients: Initiate esa at Hb=90–110 g/L based on anemia-related symptoms Asymptomatic patients: esa may be initiated at Hb<119 g/L to prevent further decline	Symptomatic patients: Hb=100–110 g/L: consider esa; Hb<100 g/L: strongly consider esa Asymptomatic patients with risk factors: consider esa
Hb target Titration	120 g/L Titrate to maintain Hb of 120 g/L or restart when levels fall to near 100 g/L	120–130 g/L Continue treatment as long as Hb≤120–130 g/L and patients show symptomatic improvement; titrate to lowest effective maintenance dose in patients reaching target Hb	120 g/L (“optimal”) In patients with a response (Hb increased by 10 g/L), titrate dose to maintain optimal Hb (120 g/L)
Dose escalation	After 4 weeks, consider dose escalation for 4–8 weeks in those who do not respond	Decision to dose-escalate cannot generally be recommended and must be individualized	Increase esa dose if no response after 4–6 weeks (depending on esa used)
Discontinuation	Continuing esa >6–8 weeks in the absence of response (<10–20 g/L Hb increase) is not beneficial	If no symptomatic improvement or Hb rise by 8–10 weeks, discontinue esa	Discontinue esa if no response at 8–12 weeks

asco/ash = American Society of Clinical Oncology/American Society of Hematology; eortc = European Organization for Research and Treatment of Cancer; nccn = National Comprehensive Cancer Network; Hb = hemoglobin.

**TABLE III:** Monitoring of erythropoiesis-stimulating agents (esas)

Epoetin alfa 12	Darbepoetin alfa	
Three times weekly and weekly dosing schedules	Weekly dosing schedule	Every-3-weeks dosing schedule
Maintenance therapy requires weekly Hb monitoring	The Canadian standard has been to monitor Hb, prior to dosing, on a weekly basis until the target Hb (120 g/L) is reached, and every 1–3 weeks thereafter	Monitor Hb, prior to dosing, at least every 3 weeks until the target Hb (120 g/L) is reached, and regularly thereafter 13

Hb = hemoglobin.

**TABLE IV:** De-escalation of erythropoiesis-stimulating agents (esas)

Epoetin alfa 12	Darbepoetin alfa 13	
Three times weekly and weekly dosing schedules	Weekly dosing schedule	Every-3-weeks dosing schedule
If Hb increases by >10 g/L in any 2-week period or if Hb>120 g/L, the dose should be reduced by 25%	If the rate of Hb increase is >10 g/L in a 2-week period or >15 g/L in a 3-week period, or when Hb>120 g/L, the dose should be reduced by 25%	If the rate of Hb increase is >15 g/L per3-week period or when Hb>120 g/L, the dose should be reduced by 40% of the previous dose
If Hb>130 g/L, the dose should be temporarily withheld until Hb<120 g/L and reinitiated at a dose 25% below the previous dose	If Hb>130 g/L, dosing should be temporarily withheld until Hb falls to 120 g/L; at that time, therapy should be reinitiated at a dose 25% below the previous dose	If Hb>130 g/L, dosing should be temporarily withheld; once Hb falls to120 g/L, therapy should be reinitiated at a dose 40% below the previous dose

Hb = hemoglobin.

**TABLE V:** Dose escalation of erythropoiesis-stimulating agents (esas) for inadequate hemoglobin (Hb) response

Epoetin alfa 12		Darbepoetin alfa 13	
Three-times-weekly dosing schedule	Weekly dosing schedule	Weekly dosing schedule	Every-3-weeks dosing schedule
If response is unsatisfactory after 4 weeks of treatment, the dose should be increased to 300 IU/kg three times weekly for 4 weeks	If response is unsatisfactory after 4 weeks of treatment, the dose should be increased to 60,000 IU weekly for 4 weeks	If no response is observed after 6 weeks of therapy, the dose can be increased to 4.5 μg/kg weekly	Dose escalation is not recommended because of an absence of additional efficacy benefit at higher dose levels for this schedule 20

**TABLE VI:** U.S. Agency for Healthcare Research and Quality Analysis summary of risk of venous thromboembolic events (vtes) by hemoglobin (Hb) level, used to stop dosing with erythropoiesis-stimulating agents 14

Target stop Hb	rr of vtes	95% ci
<120 g/L	Not estimable	NA
<130 g/L	0.70	0.29 to 1.67
<140 g/L	1.71	1.23 to 2.40
<150 g/L	1.92	1.22 to 3.02
<160 g/L	1.66	1.08 to 2.54

rr = relative risk; ci = confidence interval.

## References

[1] Ludwig H, Van Belle S, Barrett–Lee P (2004). The European Cancer Anaemia Survey (ecas): a large, multinational, prospective survey defining the prevalence, incidence, and treatment of anaemia in cancer patients. Eur J Cancer.

[2] Littlewood TJ, Bajetta E, Nortier JW, Vercammen E, Rapoport B, for the Epoetin Alfa Study Group (2001). Effects of epoetin alfa on hematologic parameters and quality of life in cancer patients receiving nonplatinum chemotherapy: results of a randomized, double-blind, placebo-controlled trial. J Clin Oncol.

[3] Witzig TE, Silberstein PT, Loprinzi CL (2005). Phase iii, randomized, double-blind study of epoetin alfa compared with placebo in anemic patients receiving chemotherapy. J Clin Oncol.

[4] Demetri GD, Kris M, Wade J, Degos L, Cella D, for the Procrit Study Group (1998). Quality-of-life benefit in chemotherapy patients treated with epoetin alfa is independent of disease response or tumor type: results from a prospective community oncology study. J Clin Oncol.

[5] Cella D (1998). Factors influencing quality of life in cancer patients: anemia and fatigue. Semin Oncol.

[6] Bohlius J, Wilson J, Seidenfeld J (2006). Recombinant human erythropoietins and cancer patients: updated meta-analysis of 57 studies including 9353 patients. J Natl Cancer Inst.

[7] Rizzo JD, Lichtin AE, Woolf SH (2002). Use of epoetin in patients with cancer: evidence-based clinical practice guidelines of the American Society of Clinical Oncology and the American Society of Hematology. Blood.

[8] Bokemeyer C, Aapro MS, Courdi A (2004). eortc guidelines for the use of erythropoietic proteins in anaemic patients with cancer. Eur J Cancer.

[9] National Comprehensive Cancer Network (nccn) (2007). Practice Guidelines in Oncology: Cancer- and Treatment-Related Anemia. Ver. 3.

[10] Rodgers GM (2006). Guidelines for the use of erythropoietic growth factors in patients with chemotherapy-induced anemia. Oncology.

[11] Bokemeyer C, Aapro MS, Courdi A (2007). eortc guidelines for the use of erythropoietic proteins in anemic patients with cancer: 2006 update. Eur J Cancer.

[12] Janssen–Ortho Inc (2006). Product Monograph: Eprex (Epoetin Alfa).

[13] Amgen Canada Inc. (2006). Aranesp (darbepoetin alfa) [product monograph].

[14] Seidenfeld J, Piper M, Bohlius J (2006). Comparative Effectiveness of Epoetin and Darbepoetin for Managing Anemia in Patients Undergoing Cancer Treatment.. Comparative Effectiveness Review No. 3..

[15] Cheung W, Minton N, Gunawardena K (2001). Pharmacokinetics and pharmacodynamics of epoetin alfa once weekly and three times weekly. Eur J Clin Pharmacol.

[16] Canon JL, Vansteenkiste J, Bodoky G (2006). Randomized, double-blind, active-controlled trial of every-3-week darbepoetin alfa for the treatment of chemotherapy-induced anemia. J Natl Cancer Inst.

[17] Couture F, Turner AR, Melosky B (2005). Prior red blood cell transfusions in cancer patients increase the risk of subsequent transfusions with or without recombinant human erythropoietin management. Oncologist.

[18] Quirt I, Kovacs M, Couture F (2006). Patients previously transfused or treated with epoetin alfa at low baseline hemoglobin are at higher risk for subsequent transfusion: an integrated analysis of the Canadian experience. Oncologist.

[19] Lyman GH, Glaspy J (2006). Are there clinical benefits with early erythropoietic intervention for chemotherapy-induced anemia? A systematic review. Cancer.

[20] Glaspy J, Henry D, Patel R (2005). Effects of chemotherapy on endogenous erythropoietin levels and the pharmacokinetics and erythropoietic response of darbepoetin alfa: a randomised clinical trial of synchronous versus asynchronous dosing of darbepoetin alfa. Eur J Cancer.

[21] Leyland–Jones B, Semiglazov V, Pawlicki M (2005). Maintaining normal hemoglobin levels with epoetin alfa in mainly nonanemic patients with metastatic breast cancer receiving first-line chemotherapy: a survival study. J Clin Oncol.

[22] Henke M, Laszig R, Rube C (2003). Erythropoietin to treat head and neck cancer patients with anaemia undergoing radiotherapy: randomized, double-blind, placebo-controlled trial. Lancet.

[23] Wright JR, Ung YC, Julian JA (2007). Randomized, double-blind, placebo-controlled trial of erythropoietin in non-small-cell lung cancer with disease-related anemia. J Clin Oncol.

[24] Crawford J, Robert F, Perry MC, Belani C, Williams D, for the Anemia Prevention in nsclc Group (2007). A randomized trial comparing immediate versus delayed treatment of anemia with once-weekly epoetin alfa in patients with non-small cell lung cancer scheduled to receive first-line chemotherapy. J Thorac Oncol.

[25] Vansteenkiste J, Pirker R, Massuti B, for the Aranesp 980297 Study Group (2002). Double-blind, placebo-controlled, randomized phase iii trial of darbepoetin alfa in lung cancer patients receiving chemotherapy. J Natl Cancer Inst.

[26] Grote T, Allen A, Castillo R (2005). Efficacy and safety analysis of epoetin alfa in patients with small cell lung cancer: a randomized, double-blind, placebo-controlled trial. J Clin Oncol.

[27] Pirker R, Ramlau R, Schuette W (2007). A phase 3 randomized, double blind, placebo-controlled study of patients with previously untreated extensive-stage small cell lung cancer (sclc) treated with platinum plus etoposide chemotherapy with or without darbepoetin alfa: PD6-3–6. J Thorac Oncol.

[28] Bohlius J, Langensiepen S, Schwarzer G (2005). Recombinant human erythropoietin and overall survival in cancer patients: results of a comprehensive meta-analysis. J Natl Cancer Inst.

[29] Henke M, Mattern D, Pepe M (2006). Do erythropoietin receptors on cancer cells explain unexpected clinical findings?. J Clin Oncol.

[30] United States, Department of Health and Human Services, Food and Drug Administration, Center for Drug Evaluation and Research, Oncologic Drugs Advisory Committee (odac) (2004). www.fda.gov/ohrms/dockets/ac/04/transcripts/4037T2.htm.

[31] Sinclair AM, Todd MD, Forsythe K, Knox SJ, Elliott S, Begley CG (2007). Expression and function of erythropoietin receptors in tumors: implications for the use of erythropoiesis-stimulating agents in cancer patients. Cancer.

[32] Canada, Health Canada (2007). Home > Drugs & Health Products > MedEffect > Advisories, Warnings & Recalls > Health Canada Endorsed Important Safety Information on Erythropoiesis-Stimulating Agents (esas): Aranesp (darbepoetin alfa) and Eprex (epoetin alfa) [Web page].

[33] Auerbach M, Ballard H, Trout JR (2004). Intravenous iron optimizes the response to recombinant human erythropoietin in cancer patients with chemotherapy-related anemia: a multicenter, open-label, randomized trial. J Clin Oncol.

[34] Vandebroek A, Gaede B, Altintas S (2006). A randomized open-label study of darbepoetin alfa administered every 3 weeks with or without parenteral iron in anemic subjects with nonmyeloid malignancies receiving chemotherapy [abstract]. J Clin Oncol.

[35] Henry DH, Dahl NV, Auerbach M (2007). Intravenous ferric gluconate significantly improves response to epoetin alfa versus oral iron or no iron in anemic patients with cancer receiving chemotherapy. Oncologist.

[36] Bellet RE, Ghazal H, Flam M (2007). A phase iii randomized controlled study comparing iron sucrose intravenously (IV) to no iron treatment of anemia in cancer patients undergoing chemotherapy and erythropoietin stimulating agent (esa) therapy. Proc Am Soc Clin Oncol.

